# A novel role of proteasomal β1 subunit in tumorigenesis

**DOI:** 10.1042/BSR20130013

**Published:** 2013-07-16

**Authors:** Fuqiang Yuan, Yana Ma, Pan You, Wenbo Lin, Haojie Lu, Yinhua Yu, Xiaomin Wang, Jie Jiang, Pengyuan Yang, Qilin Ma, Tao Tao

**Affiliations:** *Xiamen University School of Life Sciences, Xiamen, Fujian, People's Republic of China; †Institute of Biomedical Sciences and Department of Chemistry, Fudan University, Shanghai, People's Republic of China; ‡Ob/Gyn Hospital of Fudan University, Shanghai, People's Republic of China; §Xiamen Zhongshan Hospital Affiliated to Xiamen University, Xiamen, Fujian, People's Republic of China; ∥The First Hospital affiliated to Xiamen University, Xiamen, Fujian, People's Republic of China

**Keywords:** degradation, p27^Kip1^, phosphorylation, tumorigenesis, β1 subunit, CBB, Coomassie Brilliant Blue, CDK, cyclin-dependent kinase, GST, glutathione transferase, HCC, hepatocellular carcinoma, HEK-293T, HEK-293 cells expressing the large T-antigen of SV40 (simian virus 40), PKA, protein kinase A, Rb, retinoblastoma, Rfp, red fluorescent protein, RP, regulatory particle, shRNA, small hairpin RNA

## Abstract

p27^Kip1^ is a key cell-cycle regulator whose level is primarily regulated by the ubiquitin–proteasome degradation pathway. Its β1 subunit is one of seven β subunits that form the β-ring of the 20S proteasome, which is responsible for degradation of ubiquitinated proteins. We report here that the β1 subunit is up-regulated in oesophageal cancer tissues and some ovarian cancer cell lines. It promotes cell growth and migration, as well as colony formation. β1 binds and degrades p27^Kip1^directly. Interestingly, the lack of phosphorylation at Ser^158^ of the β1 subunit promotes degradation of p27^Kip1^. We therefore propose that the β1 subunit plays a novel role in tumorigenesis by degrading p27^Kip1^.

## INTRODUCTION

There are two main protein degradation machineries in eukaryotic cells, proteasomes and lysosomes [1]. The ubiquitin–26S proteasome-dependent proteolytic pathway plays important roles in many cellular processes by controlling levels of key molecules, which function in cell-cycle progression, antigen presentation, the secretory pathway and signal transduction, etc. [2]. The 26S proteasome is a large multi-subunit complex containing a 20S proteolytic CP (core particle) and a 19S RP (regulatory particle) [3]. The 20S proteasome comprises four heptameric stacked rings (α_1-7_/β_1-7_/β_1-7_/α_1-7_) and has chymotrypsin-like (β5), trypsin-like (β2) and caspase-like (β1) activities that cleave peptides after hydrophobic, basic and acidic residues, respectively [4]. The free 20S proteasome can mediate ubiquitin-independent degradation of proteins that are naturally unfolded or damaged [5,6].

The β1 subunit has caspase-like activity in the constitutively expressed mammalian proteasomal complex and is replaced by the IFNγ (interferon γ)-inducible subunit, β1i, in the immunoproteasome [7]. Most β-type subunits are synthesized as proproteins, which undergo limited proteolysis during proteasomal maturation [8–11]. The C-terminal extension of β7/Pre4 is required for the post-acidic activity mediated by the β1/Pre3 subunit and deletion of the C-terminal tail of β7/Pre4 inhibits β1/Pre3 propeptide processing and abrogation of post-acidic activity [12,13]. A mutant lacking both Blm10 and the C-terminal extension of β7/Pre4 grows extremely poorly, accumulates very high levels of precursor complexes and is impaired in β subunit maturation [14]. The processing of active eukaryotic β subunits is reported to be an ordered two-step mechanism involving autocatalysis [11,15].

Progression through the cell cycle requires the formation and activation of cyclin and CDK (cyclin-dependent kinase) complexes [16,17]. Activation of the G1phase cyclin-CDK complexes results in the phosphorylation of Rb (retinoblastoma) gene products which oppose cell-cycle progression by controlling gene expression mediated by E2F transcription factors [18]. CDKIs (CDK inhibitors), p21^cip1^, p27^Kip1^ and p15/p16^ink4^, regulate this process by inhibiting cyclin/CDK activity and phosphorylation of Rb, resulting in G1 arrest [17,19–21]. p27^Kip1^ is primarily expressed in the G0 phase of the cell cycle and regulates cell-cycle progression [22,23]. p27^Kip1^ specifically inhibits cyclin E/Cdk2 and cyclin A/Cdk2, two kinases necessary for DNA replication. When the levels of p27^Kip1^ decrease, Cdk2 is activated and cells enter S phase [24]. Regulation of cellular levels of p27^Kip1^ is therefore one of key points in cell-cycle control. Two post-translational mechanisms were proposed to be involved in p27^Kip1^ breakdown: (a) ubiquitinated p27^Kip1^ is recognized and destroyed by the proteasome [25,26], or (b) the N-terminus of non-ubiquitinated p27^Kip1^ is rapidly cleaved to remove its cyclin-binding domain, a process that is ATP-dependent with high activity in the S phase [27]. In addition, B-lymphoid cells have caspase or caspase-like activities that are inversely regulated with respect to p27^Kip1^ abundance and this activity cleaves a caspase recognition site present in p27^Kip1^ (DPSD139) [28]. Tambyrajah et al. recently used a tetra-peptide substrate, Ac-DPSD-AMC, to mimic a target cleavage site in p27^Kip1^ and traced this activity to the β1 subunit of the 20S proteasome [29]. Nevertheless, this tetra-peptide substrate may not adequately represent the p27^Kip1^ protein. Whether β1 binds and degrades p27^Kip1^ directly remains unknown.

In the 20S proteasomal phosphoproteome, Ser^157^ in murine β1 (158 in human) has been suggested to be a PKA (protein kinase A) phosphorylation site [30]. However, the biological significance of this possible phosphorylation is unknown.

We observed that the β1 subunit is up-regulated in oesophageal cancer tissues and some ovarian cancer cell lines. It promotes cell growth, colony formation and migration. Interestingly, β1 binds and degrades p27^Kip1^directly and the phosphorylation of β1 at Ser^158^ plays a key role in the degradation of p27^Kip1^. We thus present here a novel role of β1 subunit in tumorigenesis.

## MATERIALS AND METHODS

### Construction of β1 and p27^Kip1^ expression plasmids

Plasmids were constructed using standard recombinant technique as described previously [31]. The propeptide of β1 subunit (34 amino acids in the N-terminal domain of β1 zymogen) was deleted and the active sites (Thr^35^ and Thr^36^) were therefore exposed [10]. This truncated β1 was subcloned into a pGEX 4T2 vector [GST (glutathione transferase)-β1] and pDsRed1-C1 vector (Rfp-β1), whereas the full-length β1 coding sequence was subcloned into pET 28a vector (β1–His_6_). The sequences of primers and the description of plasmids were summarized in Table 1. DNA fragments encoding both β1 shRNA (small hairpin RNA) and control shRNA were subcloned into the vector pGenesil-1.0. Both siRNA (small interfering RNA) sequences of *PSMβ6* (proteasomal subunit β type 6) were used in this study: siβ1-1, aatcgagtgactgacaagctg and siβ1-2, aatgctctcgctttggccatg, respectively. A plasmid to express RNA without homology to human or mouse sequences was used as a control in silencing experiments [31].

**Table 1:** Primers used in the present study

Plasmid	5′ primer oligo	3′ primer oligo	Restriction enzyme sites
Wild-type β1			
pβ1-GST	CGCggatccACCACTATCATGGCC	CCGgaattcCGGCGGGTGGTAAAGT	BamHI/EcoRI
pβ1-His_6_	CGCggatccACCTTACTAGCTGCT	CCGgaattcGGCGGGTGGTAAAG	BamHI/EcoRI
pβ1-Rfp	CCGgaattcCACCACTATCATGGCC	CGCggatccGGCGGGTGGTAAAGTG	EcoRI/BamHI
Point-mutations in β1			
pβ1-His_6_ S158E	TGATGGTAAGGCAGGAATTTGCCATTGG	AGCCTCCAATGGCAAATTCCTGCCTTAC	
pβ1- His_6_ S158A	TGATGGTAAGGCAGGCCTTTGCCATTGG	AGCCTCCAATGGCAAAGGCCTGCCTTAC	
Wild-type p27^Kip1^			
pp27^Kip1^-GST	CGCggatccATGTCAAACGTGCGAGTGTCT	CCGctcgagTTTACGTTTGACGTCTTCTGAG	BamHI/XhoI
pp27^Kip1^- His_6_	CGCggatccATGTCAAACGTGCGAGTGTCT	CCGctcgagTTTACGTTTGACGTCTTCTGAG	BamHI/XhoI

### Cell culture

HeLa and HEK-293T [HEK-293 cells expressing the large T-antigen of SV40 (simian virus 40)] cells (obtained from A.T.C.C.) were grown in DMEM (Dulbecco's modified Eagle's medium) supplemented with 10% (v/v) FBS, 100 units/ml penicillin, 100 units/ml streptomycin at 37°C, 5% (v/v) CO_2_. Establishment of stably transfected HeLa cells was performed as described previously [32]. Cell proliferation was determined by counting the cell numbers with a haemocytometer at the indicated times after plating cells into 24-well plates for 2 h. Data represent an average of three independent experiments. As previously described [33], HeLa cells were synchronized at G0/G1 by serum starvation for 48 h and then stimulated to re-enter the cell cycle by serum re-addition. Synchronization was monitored by the Coulter EPICS XL cytometer (Beckman Coulter Inc.) using PI (propidium iodide) staining [34].

### Crystal violet staining

The plates or dishes were placed on ice and washed twice with ice-cold 1× PBS. Cells were then fixed with ice-cold 100% (v/v) methanol for 10 min. After aspiration of the methanol, 0.5% (w/v) crystal violet solution (in 25% (v/v) methanol and stored at room temperature (25°C) was added and incubated at room temperature for 10 min. After rinsing repeatedly with water, the plates were allowed to dry at room temperature and then photographed.

### Cell migration assays

Migration assays were performed using Transwells (8-μm pore size, Corning Costar) without Matrigel™, according to manufacture's instructions. Cells were allowed to migrate for 12 h at 37°C. The Transwell inserts were fixed in 10% (v/v) formalin, stained with filtered 0.5% (w/v) crystal violet in 10% (v/v) ethanol and then washed in deionized water. The non-migratory cells on the upper surfaces of the membranes were removed using cotton swabs. The membranes were air-dried and mounted for microscopy. For each chamber, the migrating cells in ten randomly chosen fields (×400) were counted.

Scratch wound assays were performed using a p200 pipette tip to create a ‘scratch.’ After washing the cells with 1 ml of growth medium, they were recultured with 1 ml of medium and photographed at regular intervals to monitor cell migration.

### Human tumour samples

Human oesophageal cancer tissue samples were obtained from Zhongshan Hospital of Xiamen University. Informed consent was obtained from the donors regarding the use of resected tumours for research purposes. The research has been carried out in accordance with the Declaration of Helsinki (2008) of the World Medical Association, that the Ethical Committee of the Institution in which the work was performed has approved it and that the subjects have given informed consent to the work.

### Immunohistochemistry

Tissue sections were prepared from formalin-fixed, paraffin-embedded specimens of human cancers. Immunohistochemical analysis was performed as described previously [35] using a mouse monoclonal antibody against β1 (#sc-100455) and Rpt3 (Proteintech Group, Inc.).

### Preparation of tissue lysates

For whole protein extracts and Western blot analysis of β1, tissues was homogenized in lysis buffer (10 mM Tris pH 7.5, 10 mM NaCl, 0.1 mM EDTA, 0.5% (v/v) Triton X-100, 0.02% (w/v) NaN_3_, 0.2 mM PMSF) containing Complete™, a protease inhibitor cocktail (# 05892791001, Roche), incubated for 30 min on ice, and then centrifuged for 30 min at 12 000 ***g***. The supernatant was saved for Western blot analysis. Protein concentrations were determined by the Bradford assay with BSA as standard [36].

### Analysis of protein–protein interactions

Both GST-tagged β1 and p27^Kip1^ and His_6_-tagged β1 and p27^Kip1^ were overexpressed by IPTG) induction in bacteria as described previously [31]. For detection of their interactions, the blot was probed with an anti-His_6_ antibody (# sc-803) or an anti-p27^Kip1^ antibody (# sc-1641) and detected by ECL. Immunoprecipitation analysis was performed as described previously [31]. In brief, 5×10^6^ cells synchronized at G0/G1 phase were lysed in lysis buffer [50 mM Hepes-NaOH (pH 7.5), 100 mM NaCl, 0.5% (v/v) Nonidet P40, 2.5 mM EDTA, 10% (v/v) glycerol, 1 mM and 1 mM PMSF]. Samples were precleared by incubation with protein G-plus-agarose beads (#sc2002) at 4°C for 1 h and then received 1 μg of a monoclonal antibody against GFP (#sc-9996), p27^Kip1^ (#sc-1641) or 1 μg of β1 (#sc-100455) along with 10 μl of protein G-plus-agarose at 4°C for 4 h. Agarose beads were washed with lysis buffer and suspended in SDS/PAGE sample buffer. After SDS/PAGE, samples were analysed by Western blotting using corresponding antibodies for β1 (#sc-100455) and p27^Kip1^ (#sc-1641). 10% of each total lysate was loaded as input and 25% of each bound sample was loaded for each Western blot analysis.

### *In vitro*/*in vivo* degradation assays

*In vitro* and *in vivo* degradation assays were partly modified from those described previously [27]. Briefly, His_6_-tagged p27^Kip1^ or its mutants and His_6_-tagged β1 protein or its mutants were expressed in *Escherichia coli* BL21 and purified using Ni Sepharose™ 6 Fast Flow. 1 μg of purified p27^Kip1^-His_6_ or its mutants and 2 μg β1-His_6_ or its mutants were mixed together at 37°C in 50 μl of degradation buffer [20 mM Hepes pH 7.2 or pH 6.5, 100 mM NaCl, 10% (w/v) sucrose, 1% (v/v) CHAPS, 10 mM DTT and 1 mM EDTA] plus 2 mM ATP and 5 mM MgCl_2_. The reactions were carried out at 37°C for different periods of time, terminated by adding SDS-gel loading buffer, and each reaction mixture was subjected to SDS/PAGE on a 12% (w/v) gel, followed by immunoblotting with either an anti-p27^Kip1^ or anti-β1 antibody.

## RESULTS

### The expression of β1 subunit is up-regulated in human tumour tissues and cells

**Figure 1:**
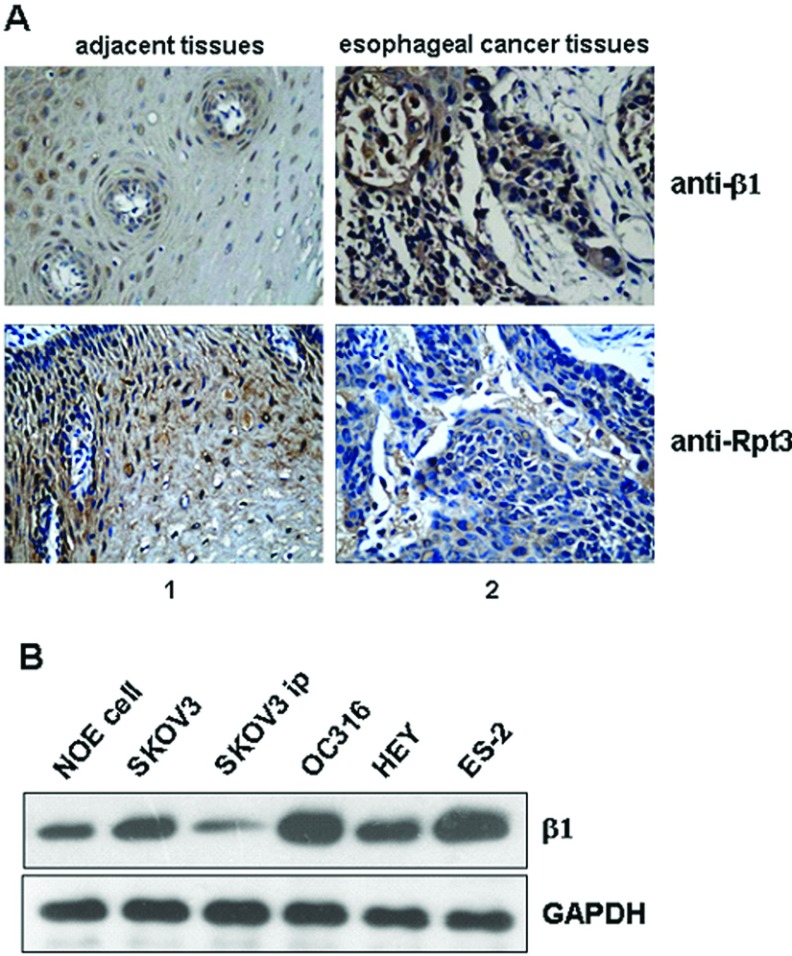
β1 subunit is up-regulated in oesophageal cancer tissues and some ovarian cancer cell lines (**A**) Immunohistochemical staining of human oesophageal cancer tissues with an anti-β1/Rpt3 antibody. Tissue sections were prepared from formalin-fixed, paraffin-embedded specimens of human cancers. Panel 1 illustrates adjacent normal tissue and panel 2 illustrates tissue of oesophageal cancer (×400). (**B**) Levels of endogenous β1in different human ovarian cancer cell lines were detected by Western blot. The level of endogenous GAPDH -(glyceraldehydes-3-phosphate dehydrogenase) was used as loading control.

As the expression of β1 subunit was observed to be up-regulated in diethylnitrosamine-treated mouse livers and in human HCC (hepatocellular carcinoma) tissues [37] and the β1 subunit has caspase activity that could degrade the key cell-cycle regulator, p27^Kip1^ [29,38], we speculated that the expression of β1 is up-regulated in other tumour tissues or cells. We therefore performed immunohistochemical staining of some paraffin-embedded oesophageal cancer tissue specimens using an antibody against β1 protein. Like in HCC samples [37], the expression of β1 is obviously up-regulated in oesophageal cancer tissues compared with its adjacent normal tissues (Figure 1A). By contrast, expression of Rpt3, a component of the 19S RP of the 26S proteasome, was not up-regulated in oesophageal cancer tissues or in its adjacent normal tissues (Figure 1A). Moreover, expression of β1 subunit was increased in several ovarian cancer cell lines (4/5 cell lines) when compared with a normal ovarian cell lines (NOE cell line) (Figure 1B).

### β1 promotes cell proliferation

**Figure 2:**
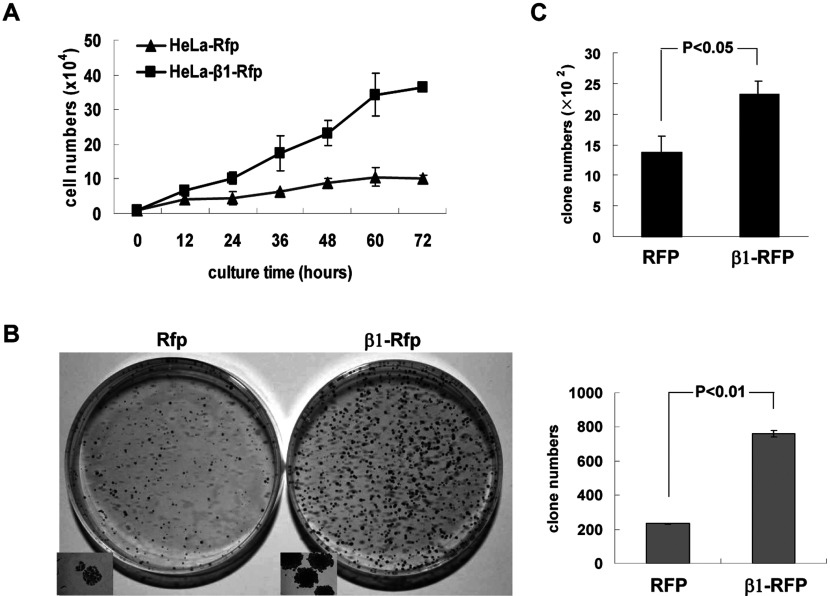
β1 promotes cell proliferation (**A**) Proliferation of HeLa cells stably expressing Rfp and β1–Rfp was determined by cell counting at the indicated times (h) 2h after cells were plated. Data represent an average of three independent experiments. (**B**) Colonies formed by HeLa cells (500 cells) stably expressing Rfp and β1–Rfp were stained with crystal violet and counted (left panel). Colonies in the small square boxes were documented by a fluorescence microscope (Nikon, ×100). Numbers of colonies are shown in the right panel. Error bars show S.D. (**C**) Plasmids expressingβ1-Rfp or Rfp were transiently transfected into HepG_2_ cells and cells were cultured with Gly^418^ at 0.5 mg/ml for 10 days before staining with crystal violet as described under the Materials and Methods section. It was clear that β1 can promote colony formation by HepG_2_ cells.

Since the expression of the β1 subunit is up-regulated in human tumour tissues and cells, it is of great interest to ask whether β1 is a potential onco-protein that promotes cell proliferation and migration. To this end, we established two stably transfected HeLa cell lines expressing the Rfp (red fluorescent protein) or β1 tagged with Rfp (β1-Rfp), respectively. As shown in Figure 2(A), cells expressing ectopic β1 grew three-time faster than cells expressing only Rfp. Moreover, overexpression of ectopic β1 significantly increased colony formation, a hallmark of transformation (Figure 2B). To see whether β1 can promote the proliferation of other cell lines, a plasmid expressing β1-Rfp or Rfp was transiently transfected into HepG_2_ cells and cells were cultured with Gly^418^ at 0.5 mg/ml for 10 days before staining with crystal violet as described under the ‘Materials and methods’ section. As shown in Figure 2(C), β1 can also promote colony formation ability of HepG_2_ cells (over 60%).

### β1 promotes cell migration

**Figure 3:**
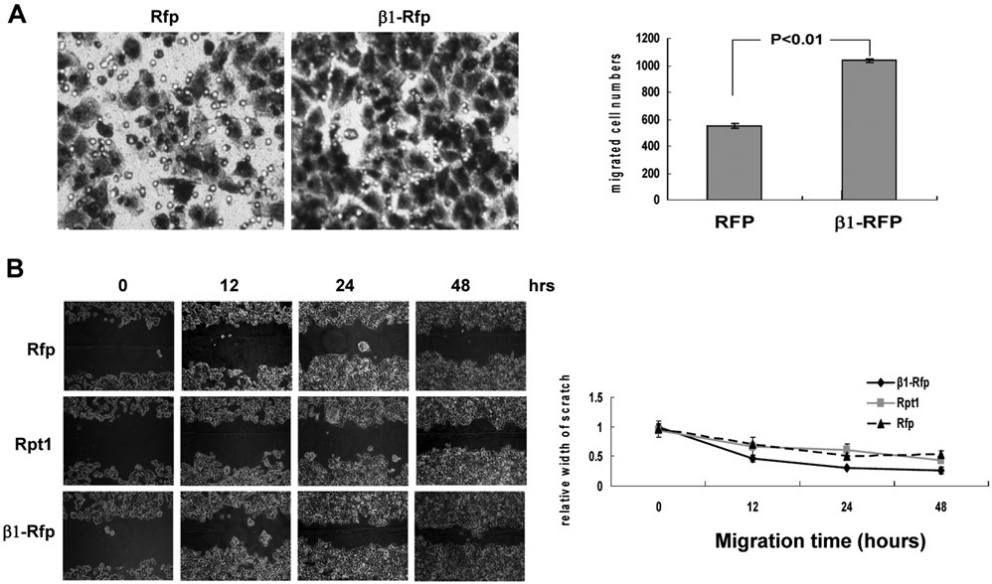
β1 promotes cell migration (**A**) Migration of HeLa cells stably expressing Rfp and β1–Rfp was observed in Transwells (8-μm pore size, Corning Costar) without Matrigel™. The membranes were air-dried, stained with crystal violet and then mounted for microscopy (left) (Nikon ×200). For each chamber the migrated cells in ten randomly chosen fields (×100) were counted (right). *Error bars* show S.D. (**B**) HEK-293T cells were transfected with plasmids expressing β1–Rfp, Rpt1 or a control vector. The cell monolayer was then ‘scratched’ with a p200 pipette tip. The dish was placed under a phase-contrast microscope and images were acquired at 0, 12, 24 and 48 h (upper panel). The width of scratch was measured and used for statistical analysis (lower panel). Error bars show S.D.

To test whether β1 can promote cell migration, Transwells were used to assay the migration ability of stably transfected HeLa cells. As shown in Figure 3(A), the number of migrated cells expressing ectopic β1 was evidently more than twice that of the control cells. Cell migration was also assessed by overexpressing β1 in HEK-293T cells with an *in vitro* scratch assay. Dynamic images of the scratch were acquired and the width of the scratch was measured as a function of time (Figure 3B). These results suggest that β1 may be a novel onco-protein, which promotes cell migration.

### β1 interacts with p27^Kip1^ *in vivo* and *in vitro*

**Figure 4:**
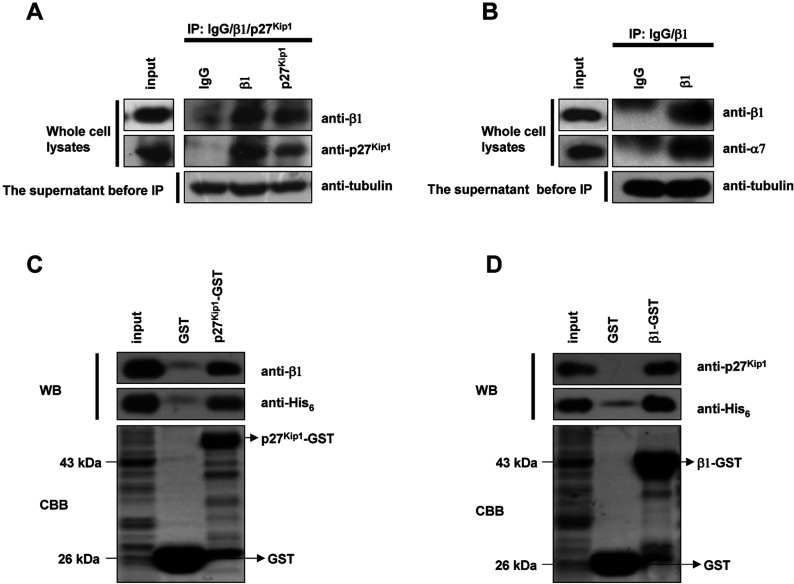
β1 interacts with p27^Kip1^ *in vivo* and *in vitro* (**A**) Immunoprecipitation analysis of the interactions between p27^Kip1^ and β1. Cell lysates from synchronized G0 HeLa cells were divided into three parts and each part was incubated with antibody (β1, GFP and p27^Kip1^ mouse monoclonal antibody) overnight and then with protein G-plus-agarose beads at 4°C for 4 h. The beads were washed three times with precipitation buffer and then treated, as above. For detection of interactions, the blot was probed with anti-β1/p27^Kip1^ rabbit polyclonal antibodies, followed by ECL. (**B**) Immunoprecipitation analysis of the interactions between β1 and the α7 subunit. For detection of this interaction, the blot was probed with anti-β1/α7 antibodies, followed by ECL. (**C**) GST-pull-down analysis of the interactions between p27^Kip1^ and β1. p27^Kip1^–GST fusion proteins were expressed by the addition of IPTG to 0.2 mM at 25°C for 4 h. Bacterial cells were lysed and the supernatants were incubated with the glutathione resin overnight and then with purified β1–His_6_ for 4 h at 4°C, followed by washing and elution. To detect interactions, the blots were probed with anti-β1/His_6_ antibodies, followed by ECL. The lower panel shows the amounts of bound GST and GST-tagged proteins by CBB (Coomassie Brilliant Blue) staining. (**D**) β1-GST fusion proteins were incubated with purified p27^Kip1^-His_6_ at 4°C for 4 h and then detected with anti-p27^Kip1^/His_6_ antibody, followed by ECL. The lower panel shows the amounts of bounded GST and GST-tagged proteins by CBB staining.

Although a recent study showed that β1 has caspase activity and can degrade p27^Kip1^ [29], the mechanism of this degradation is unknown. To investigate this process, we tested possible interactions between β1 and p27^Kip1^ using immunoprecipitation and the GST-pull-down protocols. First, we detected the possible interaction of endogenous β1 subunit and p27^Kip1^ *in vivo*. As shown in Figure 4(A), endogenous β1 and p27^Kip1^ in the precipitated complex were detected by an anti-β1 or anti-p27^Kip1^ antibody, respectively, and both precipitated β1 and p27^Kip1^ showed a remarkably efficient binding for each other. This suggests that β1 interacts with p27^Kip1^*in vivo*. As the α7 subunit is one of the constituent subunits of the 20S proteasome and it interacts with β1 *in vivo* [3,39], we next checked whether α7 was co-precipitated with β1. In Figure 4(B), the α7 subunit was found to be precipitated with β1. To test whether β1 interacts with p27^Kip1^ directly, we used a GST-pull-down assay. As shown in Figure 4(C), His_6_-tagged β1 can bind with p27^Kip1^–GST, which was detected by both anti-β1 and anti-His_6_ antibodies. Interestingly, when the β1-GST fusion protein was incubated with purified p27^Kip1^–His_6_, both anti-p27^Kip1^ and anti-His_6_ antibodies were also able to detect p27^Kip1^ (Figure 4D), suggesting that β1 and p27^Kip1^ interact with each other directly *in vitro*.

### β1 degrades p27^Kip1^ directly

**Figure 5:**
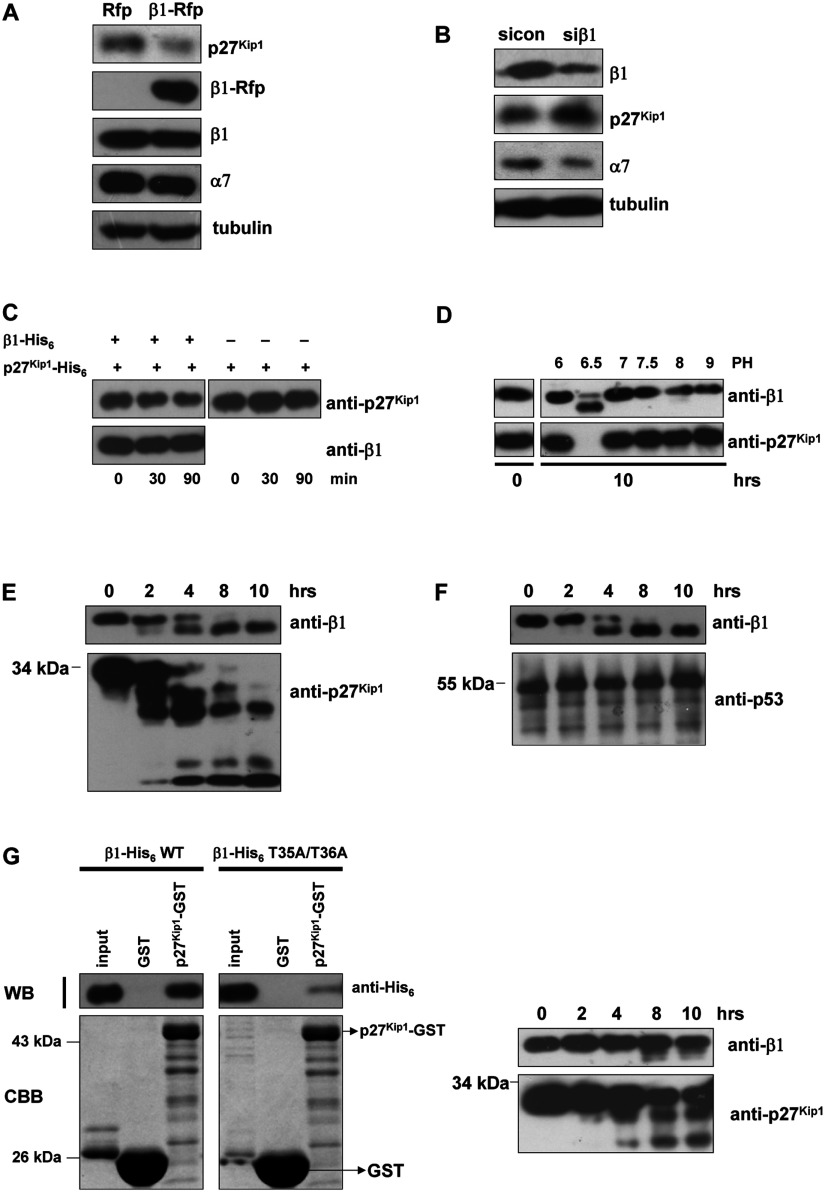
β1 is involved in the degradation of p27^Kip1^ (**A**) Stably transfected HeLa cells were synchronized at G0 phase by serum starvation for 48 h. The levels of p27^Kip1^, β1 and α7 were then determined by immunoblotting. (**B**) shRNA for β1 was transiently transfected into HeLa cells for 72 h and the levels of endogenous p27^Kip1^, β1 and α7 were detected by Western blot. (**C**) *In vitro* degradation assay for p27^Kip1^. p27^Kip1^–His_6_ and β1–His_6_ were mixed together in degradation buffer. The reactions were carried out for the indicated times and terminated by adding SDS sample buffer. Each reaction mixture was subjected to SDS/PAGE on a 12% (w/v) gel, followed by immunoblotting analysis with the anti-p27^Kip1^ and β1 antibodies. (**D**) p27^Kip1^–His_6_ and β1–His_6_ were mixed and the reaction mixtures were incubated in the buffer with the indicated pH for 10 h. Each reaction mixture was then subjected to SDS/PAGE on a 12% (w/v) gel, followed by immunoblotting analysis with anti-p27^Kip1^ and β1 antibodies. (**E**) p27^Kip1^–His_6_ and β1–His_6_ were mixed in the reaction buffer at pH 6.5 and then the mixture was incubated for the indicated period of time. The levels of β1 and p27^Kip1^ were detected by Western blot. (**F**) p53–His_6_ and β1–His_6_ were mixed in reaction buffer at pH 6.5 and this mixture was incubated for the indicated number of h. The levels of β1 and p53 were also detected by Western blot. (**G**) Only cleavable and active β1 can bind and degrade p27^Kip1^ directly. Left panel, GST-pull-down analysis for interactions between p27^Kip1^ and β1 (wide type or mutated form). The lower panel shows the amounts of bound GST and GST-tagged proteins (CBB staining). Right panel, p27^Kip1^–His_6_ and mutated β1–His_6_ (T35A/T36A) were mixed in the reaction buffer at pH 6.5 and then this mixture was incubated for the indicated periods of time. The levels of β1 and p27^Kip1^ were determined by Western blot.

We first tested whether overexpression of ectopic β1 could reduce the amount of endogenous p27^Kip1^. HeLa cells stably expressing either β1-Rfp or Rfp were synchronized at G0 phase [33]. After lysis, the amount of endogenous p27^Kip1^ was determined by immunoblotting. As shown in Figure 5(A), the level of p27^Kip1^ in cells expressing β1-Rfp was significantly lower than for cells expressing free Rfp, but the level of α7 subunit was little affected when β1 was overexpressed in the stable cell lines. To further confirm the function of β1 in degrading p27^Kip1^, plasmids expressing either control shRNAs or β1 shRNAs were expressed in HeLa cells. The amount of p27^Kip1^ in cells expressing β1 shRNAs was higher than in cells expressing the control shRNAs (Figure 5B). Interestingly, the level of the α7 subunit was also reduced when β1 was down-regulated (Figure 5B). We conclude that some essential subunits of proteasome including α7 subunit could be affected by the down-regulation of β1 and that the increased steady-state levels of p27^Kip1^ result from malfunction of proteasome [40] or down-regulating of β1.

To learn whether β1 alone can degrade p27^Kip1^, we set up an *in vitro* degradation assay that was partly modified from those described [27]. Briefly, 1 μg of purified p27^Kip1^–His_6_ and 2 μg of purified β1-His_6_ were mixed in 50 μl of degradation buffer at 37°C for increasing periods of time. We observed that the amount of p27^Kip1^ gradually decreased with time: nearly 40% was degraded after 90 min (Figure 5C). Since β1 was reported to have caspase-like activity [29,30], it is important to use further optimized conditions to study degradation of p27^Kip1^. Thus, as shown in Figure 5(D), p27^Kip1^ totally disappeared in incubations at pH 6.5 and a mobility shift of β1 band was also observed. Since β1 has a propeptide in its N-terminal domain [10], we hypothesized that β1 would be activated at pH 6.5. To test this hypothesis, p27^Kip1^–His_6_ and β1–His_6_ were mixed in the reaction buffer at pH 6.5 and for increasing periods of time (Figure 5E). The amount of p27^Kip1^ dramatically decreased with time as β1 started to self-cleave. Control His_6_-tagged p53 showed almost no change under the same conditions change (Figure 5F). The 20S proteasome is a threonine protease and its active sites are located in the N-terminal domain of β subunits. When β1 starts to self-cleave, its propeptide (34 amino acids at the N-terminal domain of the zymogen) is deleted and the active sites (Thr^35^ and Thr^36^) are therefore exposed. Deletion of the N-terminal threonine or mutating it to alanine led to inactivation of the proteasome [10]. We mutated two threonines into alanines in the N-terminal of β1–His_6_ (T35A/T36A) and found that this double mutation significantly weakened not only the interaction between β1 and p27^Kip1^ but also the ability of β1 to degrade p27^Kip1^ (Figure 5G). These results suggest that only cleavable and active β1 can bind and degrade p27^Kip1^ directly.

### Dephosphorylation of β1 at Ser^158^ enhances its ability to bind and degrade p27^Kip1^

**Figure 6:**
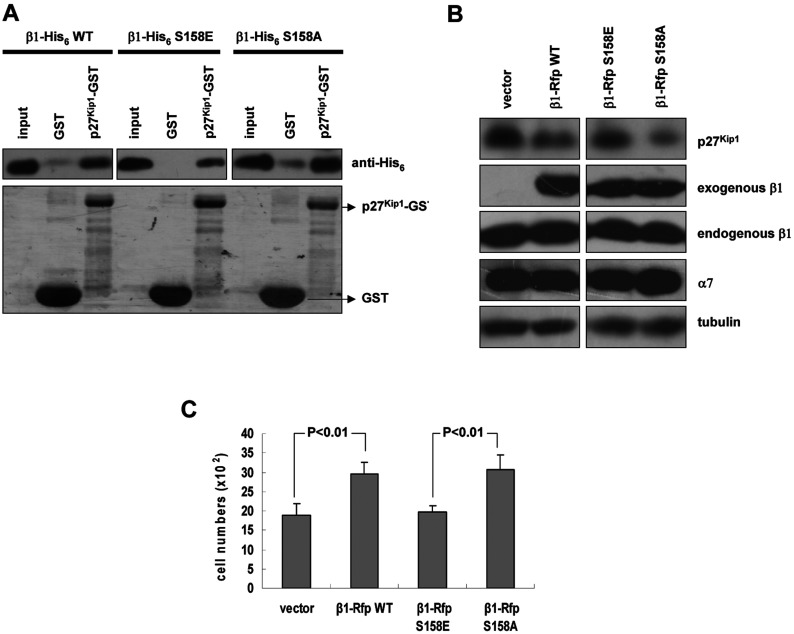
Dephosphorylation of β1 at Ser^158^ enhances its ability to bind and degrade p27^Kip1^ (**A**) Interaction assay for β1 (mutated at Ser^158^) and p27^Kip1^. p27^Kip1^–GST fusion proteins were incubated with purified β1–His_6_ wild-type (wt) or its mutant β1–His_6_ S158A or β1–His_6_ S158E at 4°C for 4 h and then detected with anti-His_6_ antibody. The lower panel shows the amounts of bound GST and GST-tagged p27^Kip1^ (CBB staining). (**B**) Endogenous p27^Kip1^, α7 and β1 were detected by immunoblotting in cells stably expressing Rfp, β1–Rfp, β1–Rfp S158A and β1–Rfp S158E, using anti-p27^Kip1^ and β1 antibodies, respectively. (**C**) Effects of Rfp-tagged β1 and β1 mutated at Ser^158^ on cell growth *in vivo*. Cell numbers were counted after plating 24 h. The data are the averages of at least three independent experiments. Error bars show S.D.

Ser^157^ in murine β1 (158 in human) has been suggested to be a PKA phosphorylation site, but its function is largely unknown [30]. Whether PKA phosphorylation of β1 regulates its role in degradation of p27^Kip1^ is an open question. To this end, two mutants of β1 were constructed: β1 S158A, to prevent the phosphorylation of β1 at Ser^158^, and β1 S158E, to mimic phosphorylation of β1 at Ser^158^ [41–44]. In an interaction assay shown in Figure 6(A), mutation of S158E greatly weakened the interaction between β1 and p27^Kip1^, but mutation of S158A shows an enhanced interaction of β1 with p27^Kip1^. To understand whether phosphorylation of β1 at Ser^158^ regulates its role in the degradation of p27^Kip1^, we compared the protein level of endogenous p27^Kip1^ in cells expressing Rfp, β1–Rfp, β1–Rfp S158A or β1–Rfp S158E, respectively and found that the amount of endogenous p27^Kip1^ in cells expressing β1 or β1 S158A was less than in cells expressing ectopic β1 S158E (Figure 6B). These results suggest that dephosphorylation of β1 at Ser^158^ enhances its ability to degrade p27^Kip1^.

As p27^Kip1^ regulates cell cycle progression, it is of great interest to ask whether phosphorylation of β1 regulates cell proliferation. To this end, the growth of cells expressing β1 S158A was compared with the growth of cells expressing β1 S158E. As shown in Figure 6(C), cells expressing β1 S158A grew faster than cells expressing β1 S158E (over 50%).

Taken together, these data suggest that phosphorylation of β1 at Ser^158^ prevents its binding and degradation of p27^Kip1^, whereas dephosphorylation of β1 at Ser^158^ increases its ability to bind and degrade p27^Kip1^, thus promoting cell proliferation.

## DISCUSSION

*In vivo* measurements show that most of tumours exhibit a significantly acidic pH when compared with normal tissues [45,46]. Increased glycolysis, a trait almost invariably observed in human cancers, confers a selective growth advantage on transformed cells because it allows them to create an environment that is differentially toxic to normal cells [45]. We found that β1 can be activated at pH 6.5 that β1 can promote cell proliferation and migration, and that it is up-regulated in oesophageal cancer tissues, HCC tissues [37] and some ovarian cancer cell lines. The weakly acidic environment of tumour tissues and transformed cells may facilitate the activation of the β1 proenzyme and therefore increase the ability of β1 to degrade its relevant substrates, such as p27^Kip1^.

CyclinE/Cdk2 activity promotes S phase transition by p27^Kip1^degradation [47]. CyclinD/Cdk4, 6 promotes cell cycle progression in early G1 to late G1 [48,49]. p27^Kip1^ inhibits the activities of these kinases directly by binding to them negatively regulates cell-cycle progression [24,27], and therefore plays a pivotal role in the control of cell proliferation [50]. The stability of p27^Kip1^ has been of recurrent interest. Several degradation mechanisms have been proposed, including ubiquitination-dependent pathways [26], the ubiquitination-independent pathways [27], a caspase-mediated pathway [51,52] and the Jab1-dependent pathway [53]. In this study, we found that β1 has a novel role in promoting cell proliferation by directly binding and degrading p27^Kip1^. As p27^Kip1^ plays a central role in controlling cell proliferation [54–56], and is intimately involved in cell death [52,57], it is not surprising that expression of p27^Kip1^ is tightly regulated by multiple mechanisms.

Overexpression of either the β1 or β5 subunit enhanced proteasomal activity and up-regulates the other proteasomal subunits [58–60]. However, Gaczynska et al. demonstrated that cells transfected with β1 and β5 subunits have elevated levels of only some proteasomal activities and that the total cellular content of proteasomes does not differ significantly between control and transfected cells [60]. The discrepancy regarding the proteasomal activities and the cellular content of proteasomes in β1- and β5-transfected HeLa and WI38/T cells is possibly caused by different cell lines [59]. In the present study, we observe that the proteasomal α7 subunit does not change significantly when β1 was overexpressed in stable HeLa cell lines. As the α7 subunit interacts with β1 *in vivo* [3,39], we infer that the structure and function of the proteasome are not greatly affected by overexpression of β1 subunit, but can be impaired upon knock-down of β1 expression. Since the relative stoichiometry of proteasomal subunits is controlled by an autoregulatory mechanism that mediates differential poly-ubiquitination and degradation of multiple subunits [61,62], we suggest that overexpression of β1 should not increase the integration of β1 into proteasomes. Nevertheless, reduction of the level of constitutive subunits (e.g. β1) would affect both the structure and the function of the proteasomal complex.

Ubiquitin-dependent degradation of p27^Kip1^ requires the poly-ubiquitination of p27^Kip1^ and other cellular proteins [Skp2 [33], Jab1 [53], etc.] and therefore needs significant time to react to external stimuli. Our data demonstrate that phosphorylation of β1 at Ser^158^ prevents its direct binding and degradation of p27^Kip1^ while dephosphorylation of β1 at Ser^158^ increases its ability to bind and degrade p27^Kip1^. PKA may play a key role in regulation of β1 and p27^Kip1^. Thus, phosphorylation and dephosphorylation of β1 could facilitate rapid adjustment of the level of p27^Kip1^. Further research will be required to address this possibility.
